# The Engineering Side of Hospital Work

**Published:** 1905-01-28

**Authors:** 


### THE ENGINEERING SIDE OF HOSPITAL WORK.

### I. INTRODUCTION.

In the following series of articles the writer proposes to discuss what he has termed the engineering side of hospital work. In hospitals, as everywhere else, engineering appliances are coming more and more into evidence. The continual growth of the principal hospitals, and the continually increasing demands in the matter of the comfort of patients and in the apparatus available for their treatment, have gradually brought engineering appliances more and more to the front, and it may be prophesied, with a reasonable amount of safety, that the development of the engineering side will go on at an increasing rate, as time rolls on, and as its advantages are more fully appreciated. As it is, in some of the leading hospitals, the engineering department has acquired a very important position, not, the writer ventures to think, the position that it will eventually occupy, but very much more important than in those hospitals where its advantages have not been appreciated. In some of the hospitals engineering appliances have been installed because they offered the only means of overcoming certain difficulties which had arisen through the development of the hospitals themselves, and only those appliances have been adopted for which there appeared to be this absolute necessity. In other hospitals, on the contrary, the full advantage of engineering appliances has been realised, not only to overcome certain difficulties, but to reduce the cost of working.

It should be understood that engineering appliances, and engineers, have two distinct functions; first, to overcome difficulties that would otherwise be insuperable, and secondly, to reduce the whole cost of working. The use of engineering appliances has enabled the troublesome problem involved in the supply of hot water at all times, to meet the constantly increasing demand, to be solved. It has enabled hospitals to deal with the awkward problems of disinfecting clothes, bedding, etc., and of their destruction. It provides an instrument in the hands of the surgeon enabling him to examine the bones of his patients without pain, and has given the surgeon numerous apparatus rendering his work easier, and more certain. In all engineering work, the first thing is to do the work effectually, and to provide, what is so essential in hospitals, that there shall be no failure, no possibility of an intermittent supply. But in all cases, when this has been accomplished, the second part of the engineer's work has been fulfilled and the total running costs have been reduced. Hospital work is now in the stage when engineering appliances, and engineers who thoroughly understand their work, can do much to ease the minds of those upon whom the responsibility for the management of the hospitals rests. Every institution which the writer visited in his recent tour round the London hospitals had two complaints, they were short of money, and they were short of space. The demands upon their space were constantly increasing, and the demands for each individual patient were constantly increasing. Public opinion, upon whose good graces the very existence of hospitals depends, is constantly increasing its demands in the matter of the treatment of the patients committed to the care of the hospital staff, while on the other hand, the public purse is not so open as it has been in times gone by in proportion to its requirements. It is here that engineering appliances will be useful, and that the skilled engineer will be able to assist the sorely-tried administrator. The skilled engineer should be able to perform for the governing bodies of the hospitals by whom he is employed the equivalent of making two blades of grass grow where one grew before. And here the writer would like to utter a word of warning. It has appeared to him, in the tour that he recently made, that administrators of hospitals, in many cases, did not appreciate the importance of engineering appliances, nor of the engineer, and what both may do to assist them in their difficulties in the future. There has always been a tendency, unfortunately, when engineering appliances have been first introduced, to minimise their importance, and to look upon their upkeep as a matter that could be handled best by a man of the grade of a mechanic. The idea prevails that the work involved in keeping the apparatus in order is dirty, and therefore a man who is not afraid of dirtying his hands is the one to employ. So long as the apparatus is small in number and in work done, and does not fulfil any very important office, this is true, to a certain extent, but the time arrives when every institution and every industry depends largely upon its engineering side, as hospitals will have to in the future, and then it is the brain of the engineer that is wanted, and that is valuable, not his hands. No engineer who is worth his salt is ashamed or afraid of dirtying his hands or his coat if necessity arises; but as in the medical profession, it is the man who knows how the hands should be used, and who has a thorough knowledge or the principles upon which each branch of engineering is based that is valuable. A few calculations that he can sometimes make in his head, and can always make at his desk, may save the management a great many pounds. It is hardly too much to say that for every £1 the thoroughly skilled engineer of modern times is paid he can save his employers £10, if he is allowed a reasonably free hand. The writer is aware that there are grave difficulties in following the advice given above. There is the scarcity of money, which operates against increasing the staff expenses, and against putting in new appliances, and there is often the scarcity of space, which operates in the same direction. But again, the skilled engineer, collaborating with the architect, should be able to go a long way towards overcoming the trouble involved in confined space; and with regard to the employment of skilled engineers, administrators will find sooner or later that they must face this question, and that they must answer it in the affirmative, or see their institutions gradually decline and die, as industries have that have not taken warning in time.

There is another warning that the writer would like to give. By skilled engineers he does not mean youths fresh from technical colleges. They will be undoubtedly cheap, in the sense that their salaries will not be high; but they will be terribly dear in the proper sense of the term. The modern engineer should have the same knowledge of his profession as the skilled surgeon has of his. The technical college student has not got this. He is not on a par, in engineering knowledge, with the surgeon who has finished his hospital course, in surgical knowledge, presuming that both work and make use of their opportunities. Later on, when the technical college student has been out in the world, and has had time to make practical experience, he may be a valuable man; but as he emerges, under present arrangements, from the college, he is worse than useless. His training has been similar to that of a surgeon, if such a thing can be imagined, who has learnt from books and models. No book that was ever written, and no model that was ever made can give experience of actual machines in actual work, any more than they can of the actual human body. The practical man, so called, is far safer than the Engineering College student; and where the practical man has worked up the principles of the sciences upon which engineering appliances are based, he is not easily beaten for safety and for skill. In the course of the articles, the writer proposes to deal with cold storage apparatus, refuse destructors, and disinfecting appliances, ventilating and heating apparatus, laundry and general steam appliances, electrical appliances of all kinds used in hospital work, and he also proposes to give a few suggestions, at the end of the series, which he hopes may lead to economy in hospital working. He takes this opportunity of acknowledging the uniform courtesy with which he was received at every one of the hospitals he recently visited, without exception, and to thank the officials of the hospitals for their kind attentions.

